# The Association of *Elastin* Gene Variants with Two Angiographic Subtypes of Polypoidal Choroidal Vasculopathy

**DOI:** 10.1371/journal.pone.0120643

**Published:** 2015-03-16

**Authors:** Suiho Yanagisawa, Yoichi Sakurada, Akiko Miki, Wataru Matsumiya, Issei Imoto, Shigeru Honda

**Affiliations:** 1 Department of Surgery, Division of Ophthalmology, Kobe University Graduate School of Medicine, 7–5–2 Kusunoki-cho, Chuo-ku, Kobe 650–0017, Japan; 2 Department of Ophthalmology, Faculty of Medicine, University of Yamanashi, 1110 Shimokato, Chuo-ku, Yamanashi 409–3898, Japan; 3 Department of Human Genetics, Institute of Health Biosciences, The University of Tokushima Graduate School, 3–18–15 Kuramoto-cho, Tokushima 770–8503, Japan; Tohoku University, JAPAN

## Abstract

**Objective:**

To compare the association of *elastin (ELN)* gene variants between two different angiographic phenotypes of polypoidal choroidal vasculopathy (PCV).

**Methods:**

We included 411 treatment-naïve PCV patients and 350 controls in the present study. PCV was classified into two phenotypes (152 Type 1 and 259 Type 2) according to the presence or absence of feeding vessels found in indocyanine-green angiography. Single nucleotide polymorphisms (SNPs) in the *ELN* region including rs868005, rs884843, rs2301995, rs13239907 and rs2856728 were genotyped using TaqMan Genotyping Assays.

**Results:**

In the allelic association analyses, rs868005 showed the strongest association with Type 2 PCV (allelic odds ratio 1.56; p = 7.4x10^-6^), while no SNP was significantly associated with Type 1 PCV. Genotype association analyses revealed the significant association of rs868005 with Type 2 PCV in log additive model and predominant model (odds ratio 1.75; p = 1.5x10^-6^ and odds ratio 1.60; p = 0.0044, respectively), but not with Type 1 PCV. These findings were further corroborated by another control group in the literature.

**Conclusions:**

There may be significantly different associations in genetic variants of *elastin* between two angiographic phenotypes of PCV.

## Introduction

Polypoidal choroidal vasculopathy (PCV) is recognized as a phenotype of neovascular age-related macular degeneration (AMD) and is considered as a type of choroidal neovascularization (CNV) [1–3]. However, previous reports suggest that some cases of PCV are a distinct vascular abnormality of choroidal vessels [4,5]. PCV often demonstrates several unique clinical manifestations throughout the natural course of the disease and in response to therapy, which are apparently different from those of typical neovascular AMD (tAMD) for reasons that are currently unknown [6].

Recent genetic association studies suggested a possible difference in the genetic susceptibility between tAMD and PCV [7–14], as well as the existence of heterogeneity among PCV cases [15–17]. We previously reported that coding variants of *elastin (ELN)*, a principle component of the elastic lamina in Bruch’s membrane, were associated with PCV but not with tAMD [8]. However, two subsequent reports with larger cohorts showed conflicting results; the *ELN* polymorphism was associated with tAMD but not with PCV [11,18]. Moreover, these studies showed an opposite (risk/protective) effect of *ELN* variants associated with tAMD. Those inconsistent results might occur in part by a deviation of allele frequencies in the control group of each study [8,11,18]. Thus, associations of *ELN* variants with tAMD and PCV remain inconclusive.

More recent studies have demonstrated different associations of rs10490924 (A69S) in *age-related maculopathy susceptibility 2 (ARMS2)* between two angiographic phenotypes of PCV [16,17]. Since the *ARMS2* polymorphism widely associates with AMD, these results indicated the existence of heterogeneity in the genetic susceptibility within PCV phenotypes. The heterogeneity may contribute to different characteristics of PCV against tAMD and might be causing inconsistencies in the association of *ELN* variants among previously reported studies, including whole PCV subtypes.

In this study, we compared the association of *ELN* gene variants between two different angiographic phenotypes of PCV.

## Materials and Methods

### Study Participants

This study was approved by the Institutional Review Board at the Kobe University Graduate School of Medicine and the University of Yamanashi, and was conducted in accordance with the Declaration of Helsinki. Written informed consent was obtained from all subjects (prior to study enrollment). All cases in this study were Japanese individuals recruited from the Department of Ophthalmology at the Kobe University Hospital and the University of Yamanashi Hospital.

**Fig 1:**
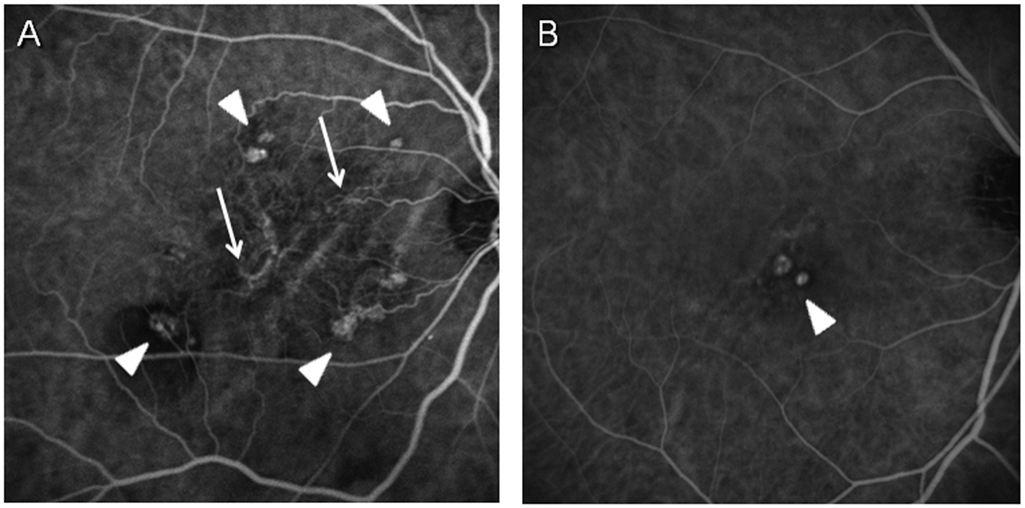
Two different angiographic subtypes of PCV. (A) Type 1 PCV shows polypoidal lesions (arrowheads) with clear branching vascular networks (arrows). (B) Type 2 PCV shows polypoidal lesions (arrowheads) without (vascular networks) or faint vascular networks.

This study included 411 consecutive treatment naïve PCV patients who consented to DNA sampling and met the criteria of PCV sub classification. Control subjects (n = 350) consisted of hospital-based volunteers from Kobe University, 50 years of age or older and were defined as individuals without macular degeneration and macular changes such as drusen or pigment abnormalities. A majority of the subjects enrolled in previous studies [8] were included in the cohort of the present study. The patients underwent ophthalmic examinations, including slit-lamp biomicroscopy of fundi, color fundus photography, optical coherence tomography. Indocyanine-green angiography (ICGA) was performed using a Heidelberg Retina Angiograph 2 (HRA2, Heidelberg Engineering, Heidelberg, Germany) or a Scanning Laser Ophthalmoscope (SLO, Rodenstock GmbH, Munich, Germany). All PCV subjects enrolled in the study met the criteria of definite cases of PCV as proposed by the Japanese Study Group of Polypoidal Choroidal Vasculopathy [19]. Briefly, ICGA showed characteristic polypoidal lesions in all PCV cases. In the present study, cases were further classified into two subtypes in accordance with the previous report [5] using ICGA findings in the early phase (within 1 minute). Briefly, 1) Type 1 PCV shows polypoidal lesions with clear branching vascular networks (feeding vessels); and 2) Type 2 PCV shows polypoidal lesions without or faint vascular networks in posterior poles on ICGA. The cases with peripapillary PCV (polypoidal lesions occurring within 1 disc diameter of the edge of the optic nerve) were excluded from the study since we suspected that they might be classified into the third category of PCV (although there is no evidence to prove this hypothesis). We also excluded cases with 1) the axial length ≧26.5mm, 2) past histories of retinal vessel occlusion, uveitis, rhegmatogenous retinal detachment or glaucoma, 3) the blockage of fluorescence due to dense subretinal hemorrhage (including hemorrhagic pigment epithelial detachment) or retinal edema and 4) ICGA images with poor resolution to distinguish branching vascular network. Typical images of each angiographic phenotype are shown in Fig. 1. The classification of the PCV phenotype was performed by more than 2 independent retinal specialists for each case under masked conditions for the genotype. Only those cases whose diagnoses were matched by all readers were included in this study.

In addition, genotype data of 336 control subjects were referred from a previous publication [18] as the second control for a replication study.

### Genotyping

Genomic DNA was extracted from peripheral blood using standard methodology. Genotyping was performed using the TaqMan single nucleotide polymorphism (SNP) Genotyping Assays or Custom TaqMan SNP Genotyping Assays (Applied Biosystems, Foster City, CA) on StepOnePlus Real-Time PCR System (Applied Biosystems) in accordance with the supplier’s recommendations. Examined SNPs were *ELN* rs868005, rs884843, rs2301995, rs13239907, rs2856728, which were examined in our previous study [8]. Briefly, SNPs were selected from the HapMap Project [20] database for the JPT (Japanese in Tokyo) population using the Tagger tool [21], with minor allelic frequencies (MAF) above 10% that correlated at D’ > 0.8 with all *ELN* SNPs of > 10% MAF.

### Splice site prediction

Two different splice-site algorithms: (BDGP: Splice Site Prediction by Neural Network) and NetGene2 (http://www.cbs.dtu.dk/services/NetGene2/), were used to predict potential splicing effects.

### Statistical Analysis

All SNPs were evaluated for the Hardy-Weinberg equilibrium using the χ^2^ test (1 degree of freedom). Allelic and genotypic frequency distributions were compared between Type 1 PCV and control groups or Type 2 PCV and control groups using the χ^2^ test with 1 or 2 degrees of freedom for the allelic and genotypic tests, respectively. Statistical power of allelic association analysis was calculated using web-based tool: (https://www.dssresearch.com/KnowledgeCenter/toolkitcalculators/statisticalpowercalculators.aspx). P-values < 0.05 were considered statistically significant.

## Results

The clinical parameters of Type 1 PCV and Type 2 PCV in Kobe and Yamanashi cohorts are listed in Table 1. The larger number of PCV cases were classified into Type 2 PCV than Type 1 PCV in either group, which showed the same trend as in a previous report [17]. There was no significant difference in age, sex among these groups except that the control cohort displayed a large female ratio in relation to the other cohorts.

**Table 1:** Data summary of the Type 1, Type 2 PCV and control subjects.

	Kobe cohort		Yamanashi cohort		Control
	Type 1 PCV	Type 2 PCV	Type 1 PCV	Type 2 PCV	
Number of subjects	102	156	48	105	350
Gender (male/female)	83/19	126/30	37/11	77/28	189/161*
Mean age ± SD (years)	74.3±7.9	73.0±7.1	73.5±8.6	72.2±8.9	70.6±7.4
Age range (years)	57–85	54–90	53–83	51–89	50–95

PCV: polypoidal choroidal vasculopathy.

*P<0.005 in comparison with each Kobe or Yamanashi Type 1 or Type 2 PCV cohort using chi-square test.

All SNPs reported in the present study did not show any significant deviations from the Hardy-Weinberg equilibrium over the entire sample (P > 0.05). S1 Table summarizes the MAF for all SNPs and the results from a single-SNP allelic association study. The MAF of rs868005, rs13239907 and rs2856728 showed nominally significant differences between Type 2 PCV and control, but the MAF of only rs868005 remained significant after the Bonferroni correction was applied. Meanwhile, no SNP showed significant difference between Type 1 PCV and control. In addition, rs868005 was significantly associated with Type 2 PCV in comparison with the control subjects in the previous publication [18] (3.5 x 10^-4^) while no association was found with Type 1 PCV. The statistical power of single-SNP association analysis at rs868005 was about 0.96 for alpha error <0.01 in the comparison between Type 2 PCV and control. To exclude the possible effect of different male/female ratio between the control and the PCV groups, multivariate logistic regression analysis was performed using age, sex, the number of minor allele at each SNP as explanatory variables. In this analysis, significant association of rs868005 was confirmed with Type 2 PCV (OR 4.56, 95%CI 1.52–2.83, p<0.0001), but not with Type 1 PCV (OR 1.45, 95%CI 0.90–2.06, p = 0.15).

Genotype association analyses in log additive model and dominant model revealed a similar tendency with allelic association analyses (S2 Table). SNPs of rs868005 showed a significant association with Type 2 PCV after the Bonferroni correction was applied, but no association was found with Type 1 PCV in those models. However, rs868005 showed significant association with both types of PCV in recessive model.

**Fig 2:**
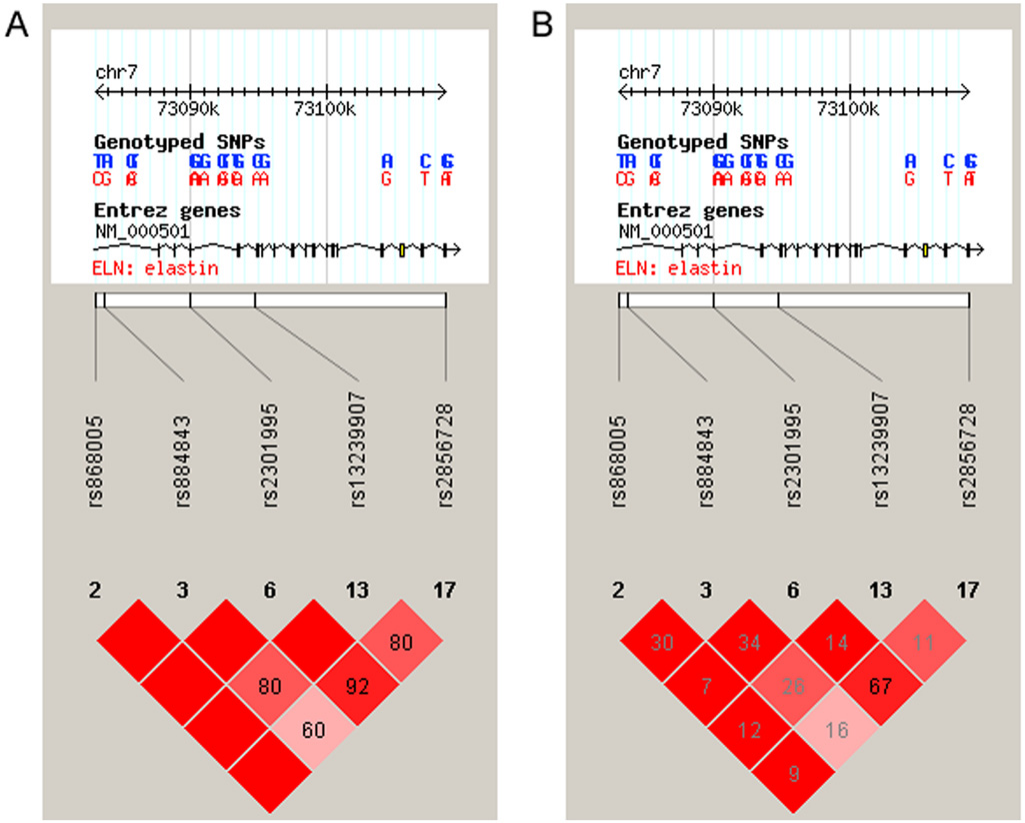
Position and linkage disequilibrium (LD) plot for 5 tag SNPs. LD values are presented by (A) D’ and (B) r^2^.

The strength of the linkage disequilibrium (LD) among SNPs selected in the present study are shown as D’ and r^2^ (Fig. 2). A haplotype-based analysis conducted with SNPs rs868005, rs884843 and rs2301995, which were evaluated in our previous study [8], further demonstrated the significant association of haplotypes consisting of rs868005/ rs884843/ rs2301995 with Type 2 PCV, but not with Type 1 PCV (S3 Table).

## Discussion

We compared the association of *ELN* gene variants between two different angiographic phenotypes of PCV and found that SNPs rs868005 showed a significant association only with Type 2 PCV, but not with Type 1 PCV in allelic association analyses and genotype association analyses with additive and dominant models.

It has not been conclusive whether *ELN* is a PCV specific susceptible gene or not since there have been inconsistent results reported among genetic association studies of this locus in the literature [8,11,18,22]. Findings in the present study have provided clues which may solve this problem. Recent studies suggest that some genetic heterogeneity may exist in PCV that is likely represented by 2 angiographic subtypes of PCV which may have a different genetic predisposition. In studies regarding *ARMS2* A69S polymorphisms, one did not find any association of *ARMS2* A69S with Type 2 PCV, whereas Type 1 PCV showed a strong association with the *ARMS2* A69S variant [16,17]. This dissociation between the two angiographic phenotypes might suggest the correlation of ARMS2 with the formation of branching vascular networks in PCV, but it also raises a possibility that Type 1 PCV and Type 2 PCV have different genetic background, hence there might be other genetic variants that differently associate between Type 1 PCV and Type 2 PCV. The associations of *ELN* variant rs868005 only with Type 2 PCV, but not with Type 1 PCV demonstrated in allelic association analyses and genotype association analyses in additive model and dominant model may provide additional evidence to support this hypothesis. However, since rs868005 showed significant association both with Type 1 and Type 2 PCV when using recessive model, a definite conclusion may not be reached only by genetic association studies. Pathophysiological effects of *ELN* variants on RPE-Bruch’s membrane-choroid complex are needed to be investigated.

*ELN* maps to chromosome 7q11 and spans 47 kb containing 34 exons. This gene encodes a protein that is one of the two components of elastic fibers. The encoded protein is rich in hydrophobic amino acids such as glycine and proline, which form mobile hydrophobic regions bounded by crosslinks between lysine residues. Multiple transcript variants encoding different isoforms have been found for this gene (elastin—Gene—NCBI). Among SNPs spanning the *ELN* region, rs868005 is located at the first intron and this SNP showed a significant association with Type 2 PCV but not with Type 1 PCV. In subgroup analyses, the allelic association results of rs868005 showed similar trends in Type 2/Type 1 odds ratio both in Kobe and Yamanashi cohorts (1.32 and 1.26, respectively). This implies that the locus including rs868005 has specific association with Type 2 PCV. Fine mapping of this locus may specify causal SNP associated with the genetic difference between Type1 PCV and Type 2 PCV. Although the functional consequence of rs868005 is unknown, a splice-site algorithm indicates that rs868005 is located by a potential cryptic splice-site, which may cause a splicing variant of the *ELN* gene and may alter the structure and function of elastin.

The underlying mechanisms which differentiate Type 1 and Type 2 PCV are largely unknown. A previous histopathological study of PCV noted that polypoidal structures were located within Bruch’s space. They were composed of clusters of dilated, thin walled blood vessels surrounded by macrophages and fibrin material [23]. Another report demonstrated large choroidal arterioles with an inner elastic layer, disruption of the inner elastic layer and arteriosclerotic changes of the vessels were identified in PCV cases [24]. A tortuous, unusually dilated venule was present adjacent to an arteriole with marked sclerotic changes, appearing to form an arteriovenous crossing. Those vessels seemed to represent native inner choroidal vessels accompanied with the findings of vascular stasis [25]. These results suggest that the polypoidal vessels in specific PCV cases represent an abnormality in the inner choroidal vasculature, which possibly correlates with Type 2 PCV. Another study reported positive immunohistochemical staining for vascular endothelial growth factor in the RPE and the vascular endothelial cells in the specimen, which suggested that the fibrovascular complex was a subretinal choroidal neovascularization [26], which might represent Type 1 PCV. In a recent study, hyalinization of the choroidal vessels and a massive exudation of fibrin and blood plasma with a change in the arteriosclerosis were observed in all of the specimens from PCV lesions [27]. The elastic layer in Bruch’s membrane might also function as a physical barrier to vessel growth from the choroid to the sub-RPE and subretinal space. In addition, elastin is not merely a structural molecule, but is a potent and specific regulator of the migration and proliferation of vascular smooth muscle cells [28]. Overall, elastin is likely critical for stabilizing the choroidal vascular structure and it is possible to play a role in the differentiation of PCV phenotypes.

Despite the results of statistical power analyses, the sample size in this study may not be large enough, which is a limitation of the present study. Since the MAF of rs13239907 and rs2856728 showed nominally significant differences between Type 2 PCV and control in the present study, a larger sample size might be needed to increase statistical power. Moreover, replication studies using different cohorts are important to confirm our results. However, the findings in the present study may support the hypothesis that the *ELN* variant differently associates between two different angiographic phenotypes in PCV.

## Supporting Information

S1 Table Comparison of single-SNP associations in different cohorts. SNP: single nucleotide polymorphism; PCV: polypoidal choroidal vasculopathy. *significant after the Bonferroni correction.
(PDF)

S2 Table Comparison of genotype association analyses. SNP: single nucleotide polymorphism; PCV: polypoidal choroidal vasculopathy; OR: odds ratio; CI: coefficient interval. *significant after the Bonferroni correction.
(PDF)

S3 Table Haplotype-based association study. Selected SNPs are rs868005/ rs884843/ rs2301995. Haplotype frequencies >0.1 in overall cohort are presented. Permutation test was performed with 10,000 iterations.
(PDF)
