# Supplementary material for: The Association of Elastin Gene Variants with Two Angiographic Subtypes of Polypoidal Choroidal Vasculopathy
Source: PLoS One. 2015 Mar 16;10(3):e0120643. doi: 10.1371/journal.pone.0120643 (PMC4361579; doi:10.1371/journal.pone.0120643)
Supplement: S1 Table — SNP: single nucleotide polymorphism; PCV: polypoidal choroidal vasculopathy. *significant after the Bonferroni correction. (PDF) [file pone.0120643.s001.pdf]

Table S1. Comparison of single-SNP associations in different cohorts

		Minor allele frequency (MAF)				Allelic association P value			
SNP ID Major/	Major/ Minor allele	Type 1 PCV (n=150)	Type 2 PCV (n=261)	Kobe Control (n=350)	Yamashiro et al [18] Control (n=336)	Type 1 vs Kobe Control	Type 2 vs Kobe Control	Type 1 vs Yamashiro et al [18] Control	Type 2 vs Yamashiro et al [18] Control
rs868005	A/G	0.25	0.32	0.21	0.23	0.12	$7.4\text{x}10^{-6}$*	0.36	$3.5\text{x}10^{-4}$*
rs884843	A/G	0.44	0.48	0.47	0.42	0.34	0.57	0.66	0.034
rs2301995	C/T	0.23	0.23	0.19	0.19	0.20	0.064	0.22	0.078
rs13239907	G/A	0.36	0.34	0.40	0.36	0.22	0.020	0.98	0.52
rs2856728	T/C	0.28	0.31	0.26	0.26	0.52	0.041	0.68	0.080

SNP: single nucleotide polymorphism; PCV: polypoidal choroidal vasculopathy. *significant after the Bonferroni correction.
