# Supplementary material for: The Association of Elastin Gene Variants with Two Angiographic Subtypes of Polypoidal Choroidal Vasculopathy
Source: PLoS One. 2015 Mar 16;10(3):e0120643. doi: 10.1371/journal.pone.0120643 (PMC4361579; doi:10.1371/journal.pone.0120643)
Supplement: S2 Table — SNP: single nucleotide polymorphism; PCV: polypoidal choroidal vasculopathy; OR: odds ratio; CI: coefficient interval. *significant after the Bonferroni correction. (PDF) [file pone.0120643.s002.pdf]

Table S2. Comparison of genotype association analyses.

SNP	Major/ Minor allele	The number of genotype (major homo/ hetero/ minor homo)			Genotype association analysis result											
					Log additive model				Dominant model				Recessive model			
		Type 1 PCV (n=150)	Type 2 PCV (n=261)	Control (n=350)	Type 1 vs Control		Type 2 vs Control		Type 1 vs Control		Type 2 vs Control		Type 1 vs Control		Type 2 vs Control	
					OR (95%CI)	P-value	OR (95%CI)	P-value	OR (95%CI)	P-value	OR (95%CI)	P-value	OR (95%CI)	P-value	OR (95%CI)	P-value
rs868005	A/G	91/42/17	130/94/37	215/122/11	1.29 (0.94-1.76)	0.12	1.76 (1.37-2.28)	1.5×10^{-6}*	1.05 (0.71-1.55)	0.81	1.63 (1.18-2.25)	0.0032*	3.92 (1.79-8.58)	6.3×10^{-4}*	5.06 (2.53-10.13)	1.3×10^{-6}*
rs884843	A/G	48/74/28	70/123/67	100/172/78	0.88 (0.67-1.16)	0.37	1.10 (0.88-1.38)	0.38	0.85 (0.56-1.29)	0.44	1.09 (0.76-1.55)	0.65	0.80 (0.49-1.30)	0.36	1.21 (0.83-1.76)	0.32
rs2301995	C/T	88/56/6	155/90/16	230/108/12	1.27 (0.91-1.78)	0.17	1.31 (0.99-1.72)	0.057	1.35 (0.91-2.00)	0.14	1.31 (0.94-1.83)	0.11	1.17 (0.43-3.19)	0.76	1.84 (0.85-3.96)	0.12
rs13239907	G/A	60/72/18	115/116/30	121/174/54	0.82 (0.62-1.10)	0.18	0.75 (0.59-0.95)	0.016	0.80 (0.54-1.18)	0.26	0.67 (0.48-0.94)	0.019	0.74 (0.42-1.32)	0.30	0.71 (0.44-1.14)	0.16
rs2856728	T/C	80/55/14	126/106/28	187/139/19	1.12 (0.82-1.52)	0.47	1.31 (1.02-1.69)	0.035	1.02 (0.69-1.50)	0.92	1.26 (0.91-1.74)	0.16	1.78 (0.87-3.65)	0.12	2.07 (1.13-3.80)	0.017

SNP: single nucleotide polymorphism; PCV: polypoidal choroidal vasculopathy; OR: odds ratio; CI: coefficient interval.

*significant after the Bonferroni correction.
