# Supplementary material for: The Association of Elastin Gene Variants with Two Angiographic Subtypes of Polypoidal Choroidal Vasculopathy
Source: PLoS One. 2015 Mar 16;10(3):e0120643. doi: 10.1371/journal.pone.0120643 (PMC4361579; doi:10.1371/journal.pone.0120643)
Supplement: S3 Table — Selected SNPs are rs868005/ rs884843/ rs2301995. Haplotype frequencies >0.1 in overall cohort are presented. Permutation test was performed with 10,000 iterations. (PDF) [file pone.0120643.s003.pdf]

Table S3. Haplotype-based association study

	SNP			Haplotype frequency				Haplotype association results	
								Type1 PCV vs Kobe control	Type2 PCV vs Kobe control
No.	Haplotype			Overall	Type1 PCV	Type2 PCV	Control	Nominal P-value (Permutation P-value)	Nominal P-value (Permutation P-value)
H1	A	A	C	0.43	0.44	0.38	0.46	0.54 (0.59)	6.1×10^{-3} (0.015)
H2	G	G	C	0.17	0.13	0.20	0.16	0.26 (0.30)	0.038 (0.068)
H3	A	G	T	0.17	0.18	0.18	0.16	0.48 (0.52)	0.41 (0.45)
H4	A	G	C	0.13	0.10	0.098	0.15	0.034 (0.17)	3.4×10^{-3} (0.056)

Selected SNPs are rs868005/ rs884843/ rs2301995. Haplotype frequencies >0.1 in overall cohort are presented.

Permutation test was performed with 10,000 iterations.
